# CiAPEX2 and CiP0, candidates of AP endonucleases in *Ciona intestinalis*, have 3′-5′ exonuclease activity and contribute to protection against oxidative stress

**DOI:** 10.1186/s41021-017-0087-7

**Published:** 2017-12-01

**Authors:** Masafumi Funakoshi, Daisuke Nambara, Yuichiro Hayashi, Qiu-Mei Zhang-Akiyama

**Affiliations:** Laboratory of Stress Response Biology, Division of Biological Sciences, Graduate School of Science, Kyoto University, Kitashirakawa-Oiwakecho, Sakyo-ku, Kyoto, 606-8502 Japan

**Keywords:** AP endonuclease, Ribosomal protein P0, 3′-5′ exonuclease activity, *Ciona intestinalis*

## Abstract

Apurinic/apyrimidinic (AP) sites are one of the most frequent DNA lesions. AP sites inhibit transcription and DNA replication, and induce cell death. AP endonucleases are key enzymes in AP site repair. Several types of AP endonucleases have been reported, such as AP endonuclease 2 (APEX2) and ribosomal protein P0 (P0). However, it is not known how the functions and roles differ among AP endonucleases. To clarify the difference of roles among AP endonucleases, we conducted biochemical analysis focused on APEX2 and P0 homologues in *Ciona intestinalis*. Amino acid sequence analysis suggested that CiAPEX2 and CiP0 are AP endonuclease homologues. Although we could not detect AP endonuclease or 3′-phosphodiesterase activity, these two purified proteins exhibited 3′-5′ exonuclease activity. This 3′-5′ exonuclease activity was sensitive to ethylenediaminetetraacetic acid (EDTA), and the efficiency of this activity was influenced by the 3′-terminus of substrate DNA. Both CiAPEX2 and CiP0 degraded not only a 5′-protruding DNA end, but also nicked DNA, which is generated through AP endonuclease 1 (APEX1) cleavage. These two genes partially complemented the growth rate of AP endonuclease-deficient *Escherichia coli* treated with hydrogen peroxide. These results indicate that 3′-5′ exonuclease activity is an evolutionarily conserved enzymatic activity of APEX2 and P0 homologues and this enzymatic activity may be important for AP endonucleases.

**Electronic supplementary material:**

The online version of this article (10.1186/s41021-017-0087-7) contains supplementary material, which is available to authorized users.

## Introduction

Apurinic/apyrimidinic (AP) sites are one of the most frequent DNA lesions, with approximately 9000 AP sites per cell produced per day in mammalian cells [1]. These DNA damages are generated via spontaneous depurination and base removal by DNA glycosylases [1–3]. If not repaired, accumulation of AP sites leads to transcription and replication blocks [4], and induces DNA mutation and cell death [5, 6]. Therefore, it is important to repair them for cell survival.

AP endonuclease, which is the core enzyme in the base excision repair (BER) pathway, plays an important role in AP site repair [7]. AP endonuclease cleaves AP sites via AP endonuclease activity, and removes a 3′-blocking end by 3′-phosphodiesterase activity, to produce a suitable form for subsequent action of the BER pathway [8, 9]. This 3′-blocking end is the product of AP site cleavage by bifunctional DNA glycosylase, which recognizes oxidized bases [8]. Many AP endonucleases are also reported to have 3′-5′ exonuclease activity [10–14]. This activity is suggested to contribute to genome integrity through stress response induction or an editing function, but the exact mechanism is not known [12, 14, 15].

AP endonuclease 1 (APEX1) has robust AP endonuclease activity and is considered to be a major AP endonuclease in human [16, 17]. However, there are also other types of enzymes implicated in AP site repair. In human, AP endonuclease 2 (APEX2) is recognized as a minor AP endonuclease because APEX2 has weak AP endonuclease activity compared with APEX1 [18]. However, APEX2 has strong 3′-phosphodiesterase and 3′-5′ exonuclease activities [14], while these activities of APEX1 are less efficient than its AP endonuclease activity [9, 19]. In addition, APEX2 null mice display growth retardation and dyslymphopoiesis [20]. Therefore, it is possible that APEX2 contributes to genome integrity in a different way than APEX1. Ribosomal protein P0 (P0) is also predicted to participate in AP site repair. Among P0 homologues, only *Drosophila melanogaster* (*D. melanogaster*) P0 homologue (DmP0) is reported to have AP endonuclease and DNase activity [21]. DmP0 is also reported to be recognized by *Homo sapiens *(*H. sapiens*) APEX1 (HsAPEX1) antibody [22]. In addition, *H. sapiens *P0 homologue is reported to interact with HsAPEX1 [23]. Considering these facts, although P0 homologues are ribosomal proteins, they are expected to adopt 3D-structures like that of AP endonuclease and participate in AP site repair.

The ascidian (sea squirt) *Ciona intestinalis* (*C. intestinalis*) is a widely used model organism in developmental biology [24]. To study the DNA repair mechanism in early development in *C. intestinalis*, our group has identified several DNA repair and sanitization genes [25–27]. In this study, to clarify the differences of roles among AP endonucleases, we focused on two candidate AP endonucleases in *C. intestinalis*, CiAPEX2 and CiP0. We herein report the results of amino acid sequence analysis, in vitro experiments using purified CiAPEX2 and CiP0, and complementation assays.

## Experimental procedures

### Bacterial strains

The genotype of *Escherichia coli* (*E. coli*) strain RPC501 (Δ*xth*, *nfo*) used in this study was described previously [28]. Unless otherwise stated, *E. coli* were grown with vigorous shaking in Luria-Bertani (LB) medium containing 100 μg/ml ampicillin at 37 °C.

### Identification and cloning of APEX2 and P0 homologue of *C. intestinalis*

By using Ghost database (http://ghost.zool.kyoto-u.ac.jp/cgi-bin/gb2/gbrowse/kh/) BLAST search, APEX2 homologue in *C. intestinalis* was searched with homology to *H. sapiens* APEX2 and the ENSCINT00000010808 clones was detected. P0 homologue in *C. intestinalis* was also detected by searching with homology to *D. melanogaster* P0 using the BLAST search, and LOC100180640 was found. Each candidate gene was amplified by PCR from a cDNA library [29] using PCR primers 5′- CGCGGATCCATGAAAATACTAACATGGAAC-3′ and 5′- CCGGAATTCTCATTTCTTTTTGTCCCATTC-3′ for CiAPEX2 and 5′- CGGGATCCATGCCTAGGGAAGACAGGAAAA-3′ and 5′- CGGAATTCTTAATCGAACAACCCGAATCCC-3′ for CiP0. The PCR products were cloned into the pGEX-4T-1 or pGEX-4T-2 vector (GE Healthcare).

### Expression and purification of GST-CiAPEX2, GST-CiP0 and tag-free CiP0

To purify CiAPEX2 and CiP0 proteins, *E. coli* strain RPC501 carrying pGEX-*CiAPEX2* or pGEX-*CiP0* was used. A single colony was inoculated into 20 ml of LB medium. An overnight culture was grown and added to 2 L of LB medium and grown until the optical density at 600 nm (OD _600_) reached 0.4 and then further incubated overnight at 20 °C in the presence of 0.01 mM isopropyl-1-thio-β-D-galactopyranoside (IPTG). After bacteria were collected, they were resuspended in buffer A (20 mM Tris-HCl pH 7.5, 1 mM dithiothreitol (DTT), 10% glycerol, 500 mM NaCl and 5 mM ethylenediaminetetraacetic acid (EDTA)). The cell suspension was sonicated and the cell lysate was centrifuged at 20,000 g at 4 °C for 30 min. The supernatant was applied to a Glutathione-Sepharose 4B column (GE Healthcare) and the purified Glutathione *S*-transferase (GST) fusion protein was eluted from the column with buffer B (20 mM Tris-HCl, pH 8.5, 150 mM NaCl, 2 mM EDTA, 1 mM DTT, 40 mM glutathione), followed by dialysis overnight at 4 °C against buffer C (30 mM Tris-HCl pH 7.5, 135 mM NaCl, 1 mM DTT, 10% glycerol, 2 mM EDTA), and stored at −80 °C until use. To purify tag-free CiP0, after the supernatant was applied to a Glutathione-Sepharose 4B column, 10 units thrombin (GE Healthcare) was then added to the column to cleave the GST at 4 °C for 24 h. After cleavage of GST by thrombin, tag-free CiP0 was eluted from the column with buffer C, and stored at −80 °C until use. Proteins were analyzed by SDS-PAGE. Using the SDS-PAGE results, protein concentration was determined using ImageJ software with the known concentration of bovine serum albumin (BSA) as a standard.

### Construction for the His-CiAPEX2 expression vector and purification of His-CiAPEX2

To prepare an expression vector for His-CiAPEX2, a CiAPEX2 cDNA fragment containing 6 histidine at its N-terminus was PCR amplified from the pGEX-4T-1-CiAPEX2 vector using PCR primers 5′-GCATCCCATGGAACATCATCATCATCATCATATGAAAATACTAACATGGAACATC-3′ and 5′-CCGGAATTCTCATTTCTTTTTGTCCCATTC-3′. The amplified fragment containing NcoI and EcoRI site at its 5′ and 3′ ends, respectively, was inserted into a pTrc99A vector. The procedure for expression and supernatant preparation was the same as GST-CiAPEX2 purification with 2 exceptions; incubation temperature after IPTG induction is 16 °C and using buffer D (20 mM Tris-HCl pH 8.0, 500 mM NaCl, 10% glycerol) containing 10 mM imidazole instead of buffer A as a resuspending buffer. The supernatant was applied to a Chelating Sepharose Fast Flow (GE Healthcare) column and the His-CiAPEX2 was eluted from the column with buffer D containing 500 mM imidazole. After dialysis against buffer E (30 mM Tris-HCl pH 7.5, 135 mM NaCl, 2 mM EDTA), fraction containing His-CiAPEX2 was loaded onto HiTrap-Q column (GE Healthcare), and eluted in Tris buffer (50 mM Tris-HCl pH 7.5, 2 mM EDTA) containing 0.235-0.32 M NaCl. The purified His-CiAPEX2 protein was dialysis against buffer C and stored at −80 °C until use.

### Detection of enzymatic activity of CiAPEX2 and CiP0

The oligonucleotides used in this study are shown in each figure. The substrate oligonucleotide was labeled with [γ-^32^P] ATP at the 5′-end using T4 polynucleotide kinase (TOYOBO, Japan). It was then annealed to the complementary oligonucleotide in buffer containing 10 mM HEPES-KOH pH 7.5 and 50 mM NaCl. In the enzymatic characterization assay, 20 nM of the ^32^P-labeled duplex DNA substrate was incubated with a recombinant protein (GST-CiAPEX2, His-CiAPEX2, GST-CiP0, or tag-free CiP0) in a 10 μl reaction mixture containing 40 mM Tris-HCl (pH 7.5), 150 mM NaCl, 8 mM MgCl_2_, 1 mM DTT, 100 μg/ml BSA. The reactions were carried out as indicated in each figure. The reaction was terminated by adding 4 μl of stop solution (95% formamide, 0.1% bromophenol blue and 20 mM EDTA). The samples were then heated at 95 °C for 5 min, immediately cooled on ice, and loaded onto 20% polyacrylamide gels, which contained Tris-borate, pH 8.3, 7 M urea and 2 mM EDTA. After electrophoresis at 1000 V for 120 min, the gels were autoradiographed using FLA-5100 (Fuji Film, Japan).

### *E. coli* Complementation assay

*E. coli* RPC501 was transformed with the pGEX plasmids carrying genes for CiPEX2 or CiP0. Overnight cultures of these bacterial transformants were diluted 100 times with fresh LB media, and allowed to grow to OD _600_ of about 0.2-0.3. Then 0.1 mM IPTG and hydrogen peroxide (H_2_O_2_) were added to the cultures and their growth was monitored.

## Results and discussion

### Identification of *Ciona intestinalis* homologues of APEX2 and P0

**Fig. 1:**
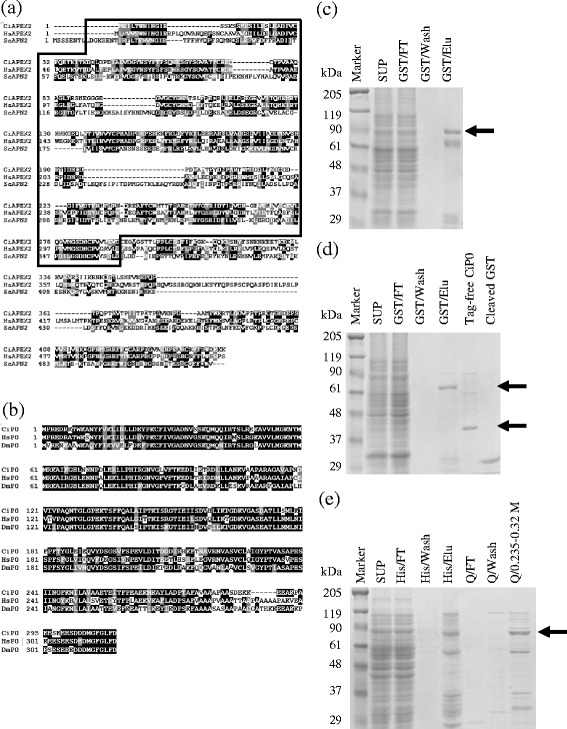
Identification of APEX2 and P0 homologues in *C. intestinalis* and purification results. **a** and **b** The amino acid sequences of *C. intestinalis* APEX2 and P0 are aligned with *H. sapiens* and *S. cerevisiae* homologues for APEX2 and *H. sapiens* and *D. melanogaster* homologues for P0. Amino acid residues are highlighted in black (identical) or grey (similar). **a** Amino acid sequence alignment among APEX2 homologues. Conserved endonuclease/exonuclease/phosphatase domain are enclosed by a black box. **b** Amino acid sequence alignment among P0 homologues. **c**-**e** Purification of GST-CiAPEX2 (**c**), GST-CiP0 (**d**), tag-free CiP0 (**d**) and His-CiAPEX2 (**e**). Each protein was purified from bacteria lysate using column chromatography. Purified proteins were electrophoresed on a 10% SDS-PAGE gel, and stained with Coomassie brilliant blue. The arrows indicate each of the purified proteins. Especially in (**d**), upper arrow indicates GST-CiP0 and lower arrow indicates tag-free CiP0. **c** and (**d** GST-CiAPEX2 (**c**), GST-CiP0 (**d**) and tag-free CiP0 (**d**) purification. The fractions are as follows: SUP, supernatant; GST/FT, flow-through fraction from Glutathione-Sepharose 4B column; GST/Wash, washed fraction by buffer A; GST/Elu, fraction eluted by 40 mM glutathione; tag-free CiP0, the product of GST-CiP0 thrombin cleavage; Cleaved GST, the byproduct of GST-CiP0 thrombin cleavage. **e** His-CiAPEX2 purification. The fractions are as follows: SUP, supernatant; His/FT, flow-through fraction from Chelating Sepharose Fast Flow; His/ Wash, washed fraction by buffer D containing 10 mM imidazole; His/Elu, fraction eluted by 0.5 M imidazole; Q/ FT, flow-through fraction from HiTrap-Q; Q/0.235-0.32 M, fractions collected in 0.235-0.32 M NaCl

Previously, our group identified an APEX1 homologue in *C. intestinalis*, and found that CiAPEX1 exhibits AP endonuclease activity and is important for early embryonic development in *C. intestinalis* (unpublished data). Previous studies showed that other AP endonucleases, such as APEX2 and P0, can function as AP endonucleases [21, 30], but the differences of the roles among these AP endonucleases are unknown. In this study, to clarify the differences of the roles and functions among AP endonucleases, we focused on the homologues of APEX2 (CiAPEX2) and P0 (CiP0) in *C. intestinalis*. By searching the *C. intestinalis* EST database from Ghost database which provides DNA sequences derived from actually expressed mRNA, clone ENSCINT00000010808 for CiAPEX2 and clone Ciad011a14 for CiP0 were detected as candidate homologues. For clone ENSCINT00000010808, predicted amino acid sequence alignments showed that CiAPEX2 shares 45 and 29% identities with *H. sapiens* and *Saccharomyces cerevisiae*(*S. cerevisiae*) homologues, HsAPEX2 and ScAPN2, respectively (Fig. 1a). HsAPEX2 and ScAPN2 have already been characterized and found to exhibit AP endonuclease, 3′-phosphodiesterase and 3′-5′ exonuclease activity [13, 14]. Application of InterPro (http://www.ebi.ac.uk/interpro/) revealed that CiAPEX2 possesses an endonuclease/exonuclease/phosphatase domain, like HsAPEX2 and ScAPN2 (Fig. 1a). These facts suggested that CiAPEX2 adopts an AP endonuclease-like conformation. Regarding clone Ciad011a14, amino acid sequence alignments showed that CiP0 shares 70 and 73% identity with *H. sapiens* and *D. melanogaster* homologues, HsP0 and DmP0, respectively (Fig. 1b). P0 is a ribosomal protein, and we could not detect an endonuclease/exonuclease/phosphatase domain in the predicted secondary structure of P0 homologues. However, since DmP0 exhibits AP endonuclease activity and is recognized by HsAPEX1 antibody [21, 22], DmP0 is expected to adopt a 3D structure similar to that of AP endonuclease. Therefore, CiP0 is also expected to be an AP endonuclease, considering its high amino acid sequence similarity to DmP0.

### Enzymatic characterization of CiAPEX2 and CiP0 in vitro

**Fig. 2:**
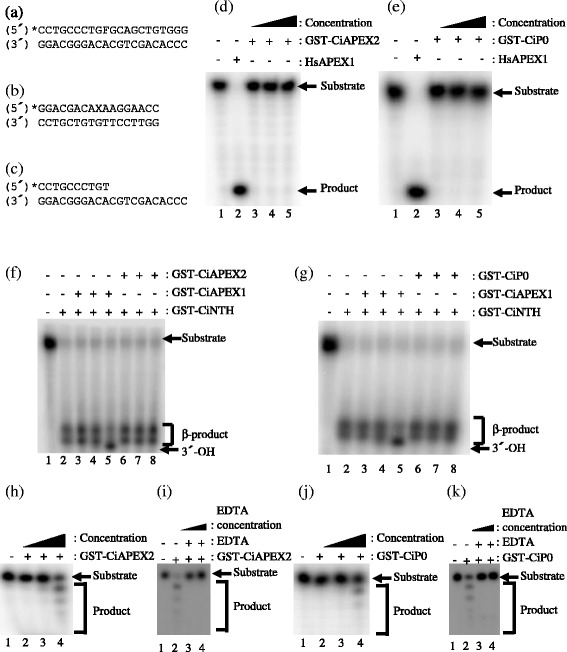
Enzymatic characterization of GST-CiAPEX2 and GST-CiP0. **a**-**c** The oligonucleotide sequences used as substrates in each experiment are displayed. In these sequences, F and X represent tetrahydrofuranyl (THF)-AP site analogue and thymine glycol (Tg). The 5′ end marked with an asterisk (*) indicates the [γ-^32^P] ATP-labeled DNA end. **d** and **e** Detection of AP endonuclease activity for GST-CiAPEX2 (**d**) and GST-CiP0 (**e**). The reaction was carried out at 28 °C for 20 min using DNA substrate shown in (**a**). Lane 1, no protein; Lane 2, 1 unit HsAPEX1(New England BioLabs); Lanes 3-5, investigated proteins. Concentration of investigated proteins were 1 nM (lane 3), 10 nM (lane 4) and 100 nM (lane 5). **f** and (**g** Detection of 3′-phosphodiesterase activity for GST-CiAPEX2 (**f**) and GST-CiP0 (**g**). The reaction was carried out at 28 °C for 20 min using DNA substrate shown in (**b**). The β-products generated by GST-CiNTH give rise to two separate bands, presumably because of Tris-adduct formation [33, 34] or isomerization of the 3′-hydroxypentenal terminus [35, 36]. Lane 1, no protein; Lane 2, GST-CiNTH alone; Lanes 3-5, GST-CiNTH and GST-CiAPEX1; Lanes 6-8, GST-CiNTH and investigated protein. Concentration of added CiNTH was constant at 1 nM. Concentration of other added proteins were as follows, 1 nM (lanes 3 and 6), 10 nM (lanes 4 and 7) and 100 nM (lanes 5 and 8). **h** and **j** Detection of 3′-5′ exonuclease activity for GST-CiAPEX2 (**h**) and GST-CiP0 (**j**). The reaction was carried out at 28 °C for 60 min using DNA substrate shown in (**c**). Lane 1, no protein; Lanes 2-4, investigated proteins. Concentration of investigated proteins were 1 nM (lane 2), 10 nM (lane 3) and 100 nM (lane 4). **i** and **k** 3′-5′ exonuclease activity of CiAPEX2 and CiP0 are dependent on metal ion. Chelating agent, EDTA, was added to the reaction buffer as indicated. Lane 1; no protein, lane 2; purified protein only, lane 3; purified protein and 10 mM EDTA, lane 4; purified protein and 20 mM EDTA

To examine the possibility that CiAPEX2 and CiP0 are AP endonucleases, we purified these proteins and investigated their enzymatic activity in vitro. To avoid endogenous AP endonuclease contamination, AP endonuclease-deficient *E. coli*, RPC501, was used as the host strain when purifying these proteins. The GST-CiAPEX2 and GST-CiP0 were induced with IPTG and purified by GSH-Sepharose column chromatography (Fig. 1c and d). Purified GST-CiP0 was further cleaved by thrombin to generate tag-free CiP0. The apparent molecular mass of each purified proteins as follows; GST-CiAPEX2 (76.7 kDa), GST-CiP0 (60.0 kDa) and tag-free CiP0 (34.0 kDa). Unfortunately, it was found that GST-CiAPEX2 was not cleaved by thrombin (data not shown). Since His tag is small and considered little effects to the enzymatic activity, we purified His-CiAPEX2 as an alternative method. His-CiAPEX2 was purified by Chelating Sepharose Fast Flow and HiTrap-Q column chromatography. The full-length band of purified His-CiAPEX2 (51.8 kDa) was indicated by arrow (Fig. 1e). Since many AP endonucleases are reported to have three enzymatic activities, AP endonuclease, 3′-phosphodiesterase, and 3′-5′ exonuclease activity, we investigated whether CiAPEX2 and CiP0 exhibited these activities in vitro. Regardless of types of tag used, we could not detect AP endonuclease or 3′-phosphodiesterase activity of all these proteins in vitro using the purified proteins (Fig. 2d-g) and Additional file 1: Figure S1(a)-(d). However, purified CiAPEX2 and CiP0 exhibited 3′-5′ exonuclease activity toward the 5′-protruding DNA substrate (Fig. 2h and j and Additional file 1: Figure S1e and f). Although GST-fused protein exhibited less 3′-5′ exonuclease activity compared with tag-free or His-tagged proteins, the properties of enzymatic activity were same among these proteins. Therefore, we used the GST-tagged proteins for further enzymatic analysis. Previous studies showed that AP endonucleases require metal ions for their enzymatic activities [31]. In the presence of a chelating agent, EDTA, the 3′-5′ exonuclease activities of CiAPEX2 and CiP0 were diminished. This result indicates that a metal ion is necessary for the enzymatic activity of CiAPEX2 and CiP0 (Fig. 2i and k).

### Characterization of 3′-5′ exonuclease activity’s substrate

**Fig. 3:**
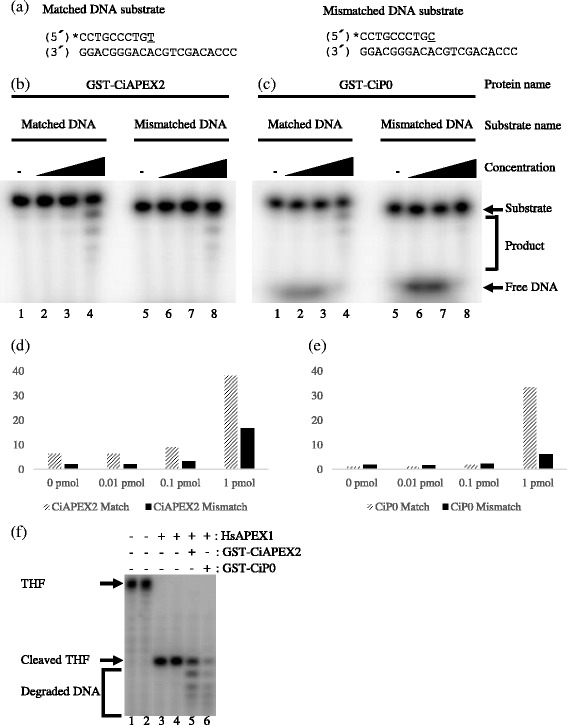
Characterization of GST-CiAPEX2 and GST-CiP0 substrate. **a** The oligonucleotide sequences used as substrates in each experiment are displayed. 5′ end marked with an asterisk (*) indicates the [γ-^32^P] ATP-labeled DNA end. In each substrate DNA sequence, underlined bases are the matched or mismatched bases focused on. **b** and **c** CiAPEX2 (**b**) and CiP0 (**c**) degraded matched DNA more efficiently than mismatched DNA. The reactions were carried out at 28 °C for 60 min using matched or mismatched DNA substrate. Lanes 1 and 5; no protein, Lanes 2-4 and 6-8; investigated proteins. Concentration of investigated proteins were 1 nM (lanes 2 and 6), 10 nM (lanes 3 and 7) and 100 nM (lanes 4 and 8). **d** Quantification results of the data shown in (**b**). **e** Quantification results of the data shown in (**c**). (**f)** CiAPEX2 and CiP0 can recognize and degrade nicked DNA, which is generated through THF cleavage by HsAPEX1. As a substrate, THF-containing DNA (Fig. 2a) was fully digested by HsAPEX1 for 60 min. Then, GST-CiAPEX2 or GST-CiP0 was added to the reaction and incubated at 28 °C for 60 min. Lane 1; no protein incubated for 60 min, Lane 2; no protein incubated for 120 min, Lane 3; only HsAPEX1 for 60 min, Lane 4; only HsAPEX1 for 120 min, Lanes 5, 6; after substrate digestion by HsAPEX1 for 60 min, 7.52 pmol GST-CiAPEX2 or 6.84 pmol GST-CiP0 was added and the mixture was incubated for 60 min

Previous studies suggested that the 3′-5′ exonuclease activity of AP endonuclease may contribute to the editing function and stress response induction [12, 14, 15]. Therefore, to investigate which of these possibilities is more likely, we next studied the substrate properties of CiAPEX2 and CiP0. From the results shown in Fig. 3b-e, we found that both proteins more efficiently degraded a protruding DNA substrate which contained a matched 3′-base pair end than a substrate with a mismatched one. This matched DNA preference is also the same for tag-free and His-tagged proteins (Additional file 2: Figure S2). These results indicate that CiAPEX2 and CiP0 are not likely to contribute to the editing function, in contrast to previous studies which suggested that 3′-5′ exonuclease activity may work as an editing function [14]. The fact that we used shorter oligonucleotide substrates than previous studies may have caused the difference between the finding of this study and previous studies. However, at least we showed that the 3′-terminus affected the degradation efficiency of the 3′-5′ exonuclease activity. The results of Fig. 3f showed that CiAPEX2 and CiP0 also recognized and degraded nicked DNA, which was generated through tetrahydrofuranyl (THF; an AP site analogue) cleavage by APEX1. This result indicates that both proteins recognize nicked DNA and produce single-stranded DNA by degrading the substrate DNA. If this reaction occurs in vivo, the stress response must be induced by Replication protein A (RPA) and ataxia-telangiectasia mutated and Rad3-related (ATR) activation [15, 32]. Therefore, CiAPEX2 and CiP0 are more likely to be involved in stress response induction than editing function.

### CiAPEX2 and CiP0 confer oxidative stress resistance to AP endonuclease-deficient *E. coli*.

**Fig. 4:**
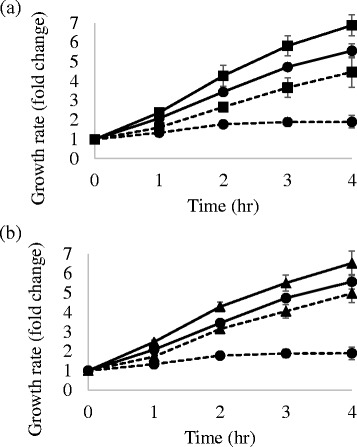
CiAPEX2 and CiP0 confer oxidative stress resistance to AP endonuclease-deficient *E. coli*. Growth rate of the *E. coli* RPC501 carrying pGEX-CiP0 (▲), pGEX-CiAPEX2 (■) or pGEX (●). Dashed lines indicate the growth rate of the *E. coli* with 300 μM H_2_O_2_ treatment and solid lines indicate the *E. coli* with no H_2_O_2_ treatment. Longitudinal axis represents the fold change relative to *E. coli* optical density at 0 h. The values represent the mean ± standard deviation (*n* = 3). **a** Complementation assay focused on CiAPEX2. **b** Complementation assay focused on CiP0

To test whether 3′-5′ exonuclease activity is an important function for AP endonuclease, we carried out complementation assays using *E. coli* RPC501 (Δ*xth*, *nfo*). After H_2_O_2_ treatment, the growth rate of RPC501 carrying the vector plasmid declined compared with the growth rate of untreated RPC501 carrying the vector plasmid. However, RPC501 carrying the pGEX-CiAPEX2 or pGEX-CiP0 plasmid and treated with H_2_O_2_ showed partial recovery of the growth rate (Fig. 4). Although it is possible that CiAPEX2 and CiP0 have weak AP endonuclease or 3′-phosphodiesterase activity below the detection limits of our in vitro experiments using purified proteins, this result suggests that the 3′-5′ exonuclease activity, which is evolutionally conserved among *S. cerevisiae* (fungus), *C. intestinalis* (chordate), *D. melanogaster* (arthropod) and *H. sapiens* (mammals) may be the important function for AP endonuclease (Fig. 4).

## Conclusions and implications

We revealed here that the 3′-5′ exonuclease activity is an evolutionarily conserved enzymatic activity of APEX2 and P0 homologues, and this enzymatic activity may be important for AP endonuclease function. We also revealed that both proteins are more likely to be involved in stress response than in editing function. Since *C. intestinalis* is the widely used model organism living in the sea, studies using *C. intestinalis* focused on DNA repair will be valuable for assessing the effects of marine pollution on organisms living in the sea.

## Additional files

Additional file 1: Figure S1. Enzymatic characterization of His-CiAPEX2 and tag-free CiP0. ((a) and (b)) Detection of AP endonuclease activity for His-CiAPEX2 (a) and tag-free CiP0 (b). The reaction was carried out at 28 °C for 20 min using DNA substrate shown in Fig. 2a. Lane 1, no protein; Lane 2, 1 unit HsAPEX1; Lanes 3-5, investigated proteins. Concentration of investigated proteins were 1 nM (lane 3), 10 nM (lane 4) and 100 nM (lane 5). (c) Detection of 3′-phosphodiesterase activity for His-CiAPEX2. The reaction was carried out at 28 °C for 10 min (lanes 3, 5, 8 and 10) or 20 min (lanes 4, 6, 9 and 11) using DNA substrate shown in Fig. 2b. The β-products generated by GST-CiNTH give rise to two separate bands, presumably because of Tris-adduct formation [33, 34] or isomerization of the 3′-hydroxypentenal terminus [35, 36]. Lane 1, no protein; Lane 2 and 7, GST-CiNTH alone; Lanes 3-6, GST-CiNTH and GST-CiAPEX1; Lanes 8-11, GST-CiNTH and His-CiAPEX2. Concentration of added CiNTH was constant at 1 nM. Concentration of other added proteins were as follows, 10 nM (lanes 3, 4, 8 and 9) and 100 nM (lanes 5, 6, 10 and 11). (d) Detection of 3′-phosphodiesterase activity for tag-free CiP0. The reaction was carried out at 28 °C for 20 min using DNA substrate shown in Fig. 2b. Lane 1, no protein; Lane 2, GST-CiNTH alone; Lanes 3-5, GST-CiNTH and GST-CiAPEX1; Lanes 6-8, GST-CiNTH and tag-free CiP0. Concentration of added CiNTH was constant at 1 nM. Concentration of other added proteins were 1 nM (lanes 3 and 6), 10 nM (lanes 4 and 7) and 100 nM (lanes 2, 5 and 8). ((e) and (f)) Detection of 3′-5′ exonuclease activity for HIS-CiAPEX2 (e) and tag-free CiP0 (f). The reaction was carried out at 28 °C for 60 min using DNA substrate shown in Fig. 2c. Lane 1, no protein; Lanes 2-4, investigated proteins. Concentration of investigated proteins were 1 nM (lane 2), 10 nM (lane 3) and 100 nM (lane 4). (PPTX 140 kb)

Additional file 2: Figure S2. Characterization of His-CiAPEX2 and tag-free CiP0 substrate. ((a) and (b)) CiAPEX2 (a) and CiP0 (b) degraded matched DNA more efficiently than mismatched DNA. The reactions were carried out at 28 °C for 60 min using DNA substrate shown in Fig. 3a. Lane 1 and 5; no protein, Lanes 2-4 and 6-8; investigated proteins. Concentration of investigated proteins were 1 nM (lanes 2 and 6), 10 nM (lanes 3 and 7) and 100 nM (lanes 4 and 8). (c) Quantification results of the data shown in (a). (d) Quantification results of the data shown in (b). (PPTX 82 kb)

## Abbreviations

AP site: Apurinic/apyrimidinic site
APEX1: AP endonuclease 1
APEX2: AP endonuclease 2
ATR: ataxia-telangiectasia mutated and Rad3-related
BER: base excision repair
BSA: bovine serum albumin
*C. intestinalis*: *Ciona intestinalis, ascidian*
DTT: Dithiothreitol
*E. coli*: *Escherichia coli*
EDTA: Ethylenediaminetetraacetic acid
EST: expressed sequence tag
GST: Glutathione *S*-transferase
H_2_O_2_: hydrogen peroxide
IPTG: Isopropyl-D-1-thiogalactopyranoside
LB: Luria-Bertani
OD _600_: optical density at 600 nm
P0: ribosomal protein P0
RPA: Replication protein A
THF: Tetrahydrofuran
