# Supplementary material for: CiAPEX2 and CiP0, candidates of AP endonucleases in Ciona intestinalis, have 3′-5′ exonuclease activity and contribute to protection against oxidative stress
Source: Genes Environ. 2017 Dec 1;39:27. doi: 10.1186/s41021-017-0087-7 (PMC5709841; doi:10.1186/s41021-017-0087-7)
Supplement: Supplementary file 1 — Enzymatic characterization of His-CiAPEX2 and tag-free CiP0. ((a) and (b)) Detection of AP endonuclease activity for His-CiAPEX2 (a) and tag-free CiP0 (b). The reaction was carried out at 28 °C for 20 min using DNA substrate shown in Fig. 2a. Lane 1, no protein; Lane 2, 1 unit HsAPEX1; Lanes 3-5, investigated proteins. Concentration of investigated proteins were 1 nM (lane 3), 10 nM (lane 4) and 100 nM (lane 5). (c) Detection of 3′-phosphodiesterase activity for His-CiAPEX2. The reaction was carried out at 28 °C for 10 min (lanes 3, 5, 8 and 10) or 20 min (lanes 4, 6, 9 and 11) using DNA substrate shown in Fig. 2b. The β-products generated by GST-CiNTH give rise to two separate bands, presumably because of Tris-adduct formation [33, 34] or isomerization of the 3′-hydroxypentenal terminus [35, 36]. Lane 1, no protein; Lane 2 and 7, GST-CiNTH alone; Lanes 3-6, GST-CiNTH and GST-CiAPEX1; Lanes 8-11, GST-CiNTH and His-CiAPEX2. Concentration of added CiNTH was constant at 1 nM. Concentration of other added proteins were as follows, 10 nM (lanes 3, 4, 8 and 9) and 100 nM (lanes 5, 6, 10 and 11). (d) Detection of 3′-phosphodiesterase activity for tag-free CiP0. The reaction was carried out at 28 °C for 20 min using DNA substrate shown in Fig. 2b. Lane 1, no protein; Lane 2, GST-CiNTH alone; Lanes 3-5, GST-CiNTH and GST-CiAPEX1; Lanes 6-8, GST-CiNTH and tag-free CiP0. Concentration of added CiNTH was constant at 1 nM. Concentration of other added proteins were 1 nM (lanes 3 and 6), 10 nM (lanes 4 and 7) and 100 nM (lanes 2, 5 and 8). ((e) and (f)) Detection of 3′-5′ exonuclease activity for HIS-CiAPEX2 (e) and tag-free CiP0 (f). The reaction was carried out at 28 °C for 60 min using DNA substrate shown in Fig. 2c. Lane 1, no protein; Lanes 2-4, investigated proteins. Concentration of investigated proteins were 1 nM (lane 2), 10 nM (lane 3) and 100 nM (lane 4). (PPTX 140 kb) [file 41021_2017_87_MOESM1_ESM.pptx]

## Slide 1
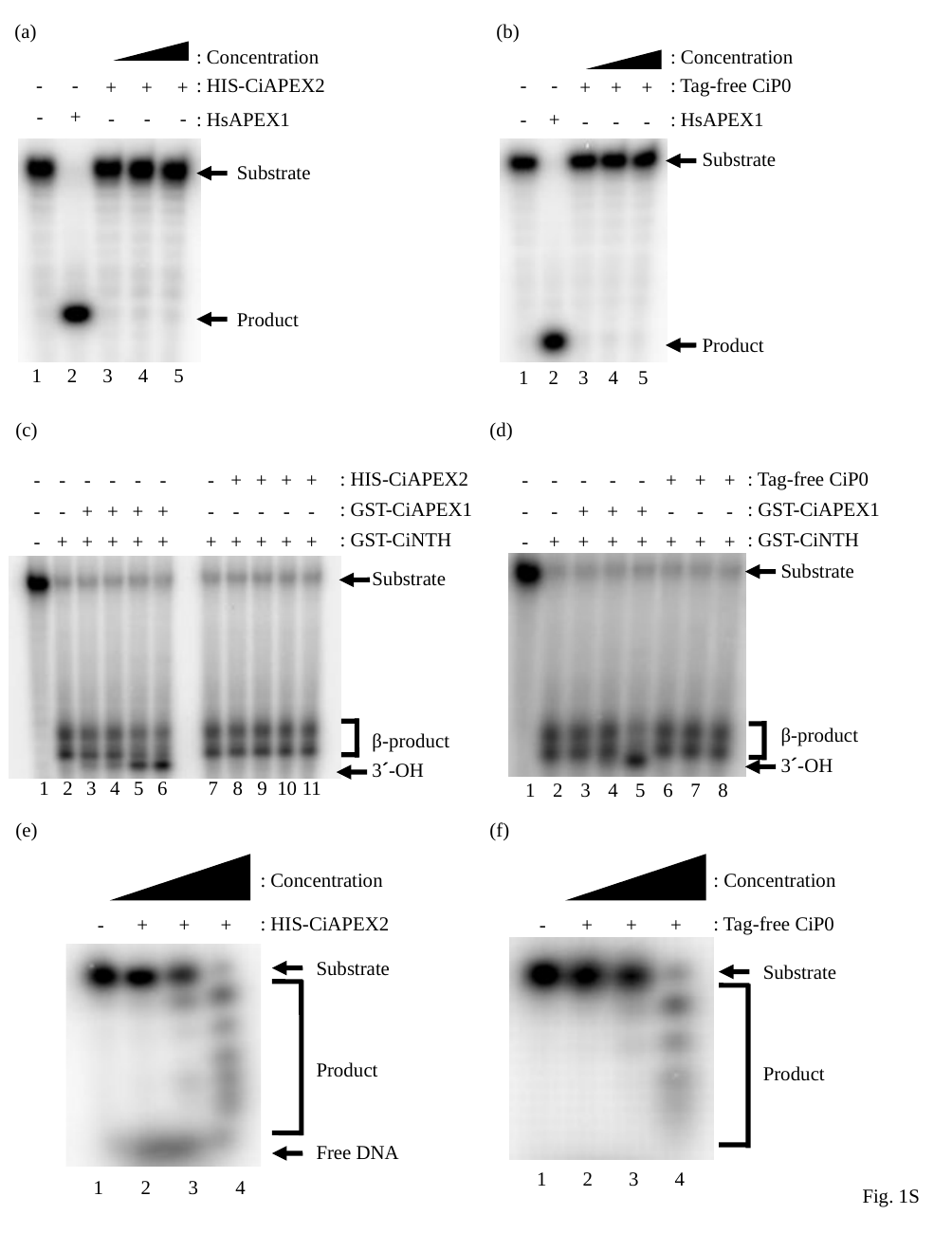

(a)
(b)
: Concentration
: Concentration
-
-
+
+
+
-
-
+
+
+
: HIS-CiAPEX2
: Tag-free CiP0
-
+
-
-
-
-
+
-
-
-
: HsAPEX1
: HsAPEX1
Substrate
Substrate
Product
Product
1
2
3
4
5
1
2
3
4
5
(c)
(d)
-
-
-
-
-
-
-
+
+
+
+
-
-
+
+
+
+
-
-
-
-
-
-
+
+
+
+
+
+
+
+
+
+
: HIS-CiAPEX2
: GST-CiAPEX1
: GST-CiNTH
-
-
-
-
-
+
+
+
-
-
+
+
+
-
-
-
-
+
+
+
+
+
+
+
: Tag-free CiP0
: GST-CiAPEX1
: GST-CiNTH
1
2
3
4
5
6
7
8
Substrate
β-product
3´-OH
1
2
3
4
5
6
7
8
9
10
11
Substrate
β-product
3´-OH
(e)
(f)
-
+
+
+
: Concentration
: HIS-CiAPEX2
Substrate
Product
Free DNA
1
2
3
4
-
+
+
+
: Concentration
: Tag-free CiP0
Substrate
Product
1
2
3
4
Fig. 1S
