# Supplementary material for: CiAPEX2 and CiP0, candidates of AP endonucleases in Ciona intestinalis, have 3′-5′ exonuclease activity and contribute to protection against oxidative stress
Source: Genes Environ. 2017 Dec 1;39:27. doi: 10.1186/s41021-017-0087-7 (PMC5709841; doi:10.1186/s41021-017-0087-7)
Supplement: Supplementary file 2 — Characterization of His-CiAPEX2 and tag-free CiP0 substrate. ((a) and (b)) CiAPEX2 (a) and CiP0 (b) degraded matched DNA more efficiently than mismatched DNA. The reactions were carried out at 28 °C for 60 min using DNA substrate shown in Fig. 3a. Lane 1 and 5; no protein, Lanes 2-4 and 6-8; investigated proteins. Concentration of investigated proteins were 1 nM (lanes 2 and 6), 10 nM (lanes 3 and 7) and 100 nM (lanes 4 and 8). (c) Quantification results of the data shown in (a). (d) Quantification results of the data shown in (b). (PPTX 82 kb) [file 41021_2017_87_MOESM2_ESM.pptx]

## Slide 1
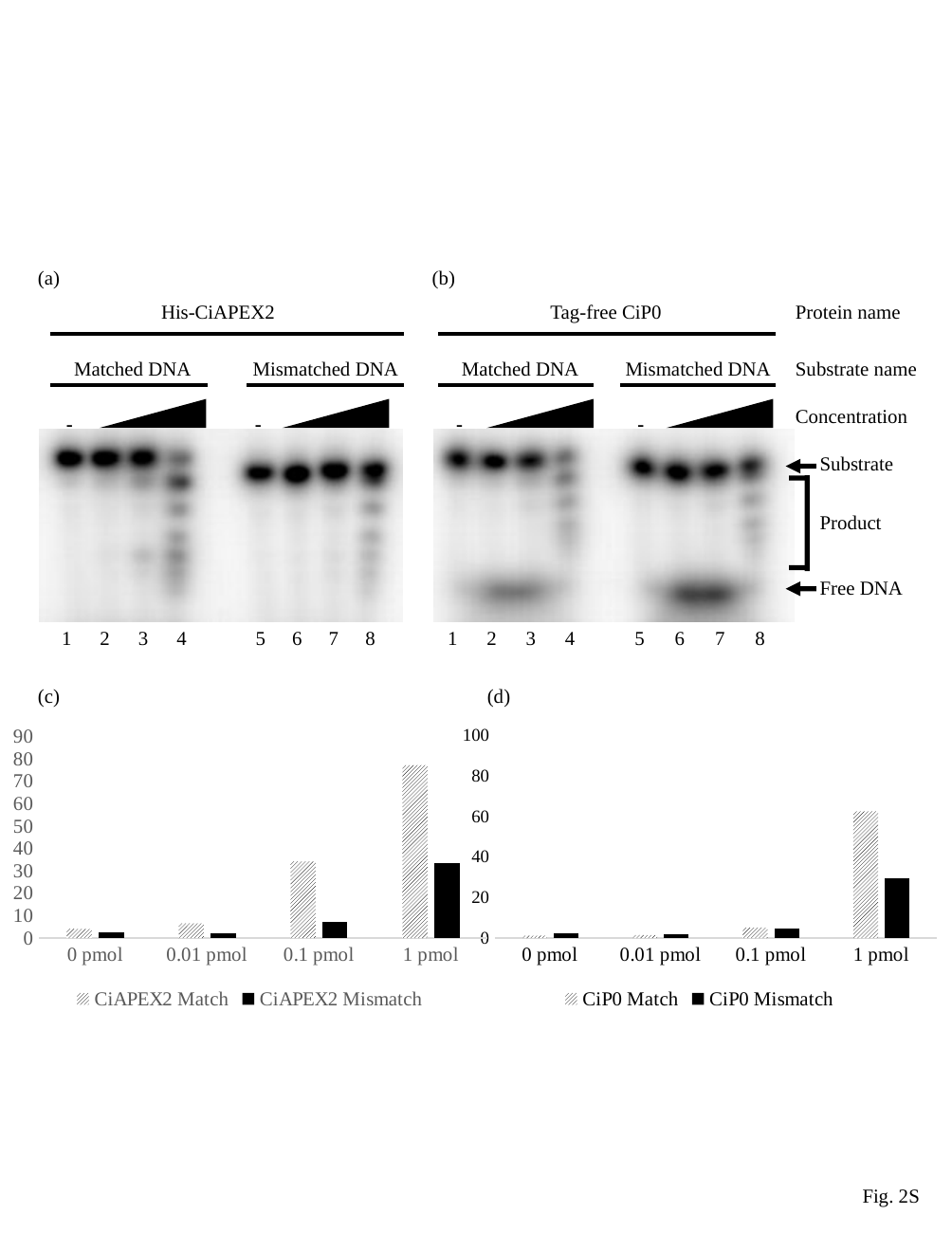

(a)
(b)
His-CiAPEX2
Tag-free CiP0
Protein name
Substrate name
Concentration
Matched DNA
Mismatched DNA
Matched DNA
Mismatched DNA
-
-
-
-
1
2
3
4
5
6
7
8
1
2
3
4
5
6
7
8
Substrate
Product
Free DNA
(c)
(d)
### Chart
| Category | CiAPEX2 Match | CiAPEX2 Mismatch |
|---|---|---|
| 0 pmol | 4.1274676267246075 | 2.388391182169113 |
| 0.01 pmol | 6.645743488986633 | 1.984762015361294 |
| 0.1 pmol | 34.24249890310018 | 7.228745632260153 |
| 1 pmol | 77.21914076959249 | 33.57750568465404 |
### Chart
| Category | CiP0 Match | CiP0 Mismatch |
|---|---|---|
| 0 pmol | 1.2722645769057535 | 2.312529762902651 |
| 0.01 pmol | 1.5114209602320732 | 2.0448490386663893 |
| 0.1 pmol | 5.195772669240027 | 4.7523248316142475 |
| 1 pmol | 62.62016807798759 | 29.684328039844054 |Fig. 2S
